# Knowledge and attitude of general population towards climate change and its impact on health in Ismailia Governorate, Egypt

**DOI:** 10.1186/s42506-024-00162-y

**Published:** 2024-07-01

**Authors:** Sarah M. Hussein, Bassma A. Ibrahim

**Affiliations:** Department of Public Health, Community Medicine, Environmental Medicine, and Occupational Medicine, Faculty of Medicine, Suez Canal University, Ismailia, Egypt

**Keywords:** Climate change, Knowledge, Attitude, Ismailia, Gender, Education

## Abstract

**Background:**

Recently, climate change (CC) has garnered significant global attention. It has emerged as one of the most pressing environmental issues, resulting in a multitude of adverse impacts on human well-being and health. This study aims to assess the knowledge and attitude of the general population in Ismailia Governorate, Egypt, about CC and its impact on health, identify factors affecting the general population’s knowledge about CC, and highlight methods to solve this problem.

**Methods:**

A cross-sectional study was carried out on the general population in Ismailia governorate, Egypt. A snowball sample of participants (*n* = 150) was enrolled in the study by distributing an online Google form containing a structured self-administered questionnaire.

**Results:**

The participants had an average knowledge score of 27.42 ± 14.42, with 60% considered knowledgeable. About 54% knew the main cause of CC. Around 75% were aware of the environmental impacts of CC, and 69.3% knew about the effects on human health. Based on the questionnaire’s results, 76.7% of respondents believed that increasing afforestation helps in the mitigation of CC and 77.3% believed that governments bear the responsibility for CC. Approximately 85% regarded increasing green spaces as an effective method to reduce CC on the country level. On an individual level, usage of energy-saving products was the most-favored option chosen by participants to help in reducing CC (82%). Gender, education level, and place of residence were significant factors affecting knowledge about CC.

**Conclusions:**

Over 50% of the participants were knowledgeable about CC and the role of human activities in CC. Therefore, public awareness campaigns utilizing prominent media such as television and social media should be launched to improve CC literacy. These campaigns should be more directed at males, and people with lower levels of education and who live in rural areas in Ismailia Governorate, Egypt.

**Supplementary Information:**

The online version contains supplementary material available at 10.1186/s42506-024-00162-y.

## Introduction

Climate change (CC) has become a global issue lately. It has emerged as one of the most serious environmental concerns. CC has a wide range of negative impacts on human life and health, particularly in developing countries [1]. CC refers to the alteration of the climate system over an extended period and a vast geographical area, resulting from either natural or human-induced processes [2]. CC is evidenced by global warming, catastrophic wind events, and severe weather conditions like heat waves, floods, and prolonged droughts. While natural processes contribute minimally to CC, human activities remain the most significant factor. Notably, greenhouse gases are the primary drivers of CC, primarily due to economic and population growth [1].

The adverse effects of CC are already evident on both physical and mental well-being. It can lead to morbidity and mortality due to more frequent occurrences of extreme weather events such as heatwaves, storms, and floods. Additionally, it can cause an increase in zoonotic diseases, as well as food, water, and vector-borne diseases [3]. Egypt is amongst the nations that are highly vulnerable to CC, whereby the anticipated rise in temperature will inevitably affect agricultural production and human well-being [4].

Many studies have addressed the issues of CC; a multi-national study conducted on samples from the United States, Canada, and Malta revealed that 30% of Americans, 50% of Canadians, and about two-thirds of Maltese, said that people are harmed by CC [5]. In addition, a Nigerian study showed that around 50% of rural farmers have a good level of awareness [6]. Also, an Indian study carried out in an urban community showed that 91.7% of participants believed that the global climate is changing [7]. Recently, in an Egyptian study by Salem et al. [8], a noteworthy percentage of participants demonstrated awareness about CC (71.1%). Although CC impacts the environment globally and poses a threat to human life, research assessing CC knowledge among Egyptians is scarce. This research aims to evaluate the knowledge and attitude of the general population in Ismailia governate about CC and its impact on health. The study also aims to identify the factors that influence the general public’s knowledge concerning CC. This will enable the identification of gaps in Egyptians’ perceptions of CC and suggest changes that can contribute to CC adaptation and mitigation measures.

## Methods

### Study design

It is a cross-sectional study carried out from 1 October 2022 to the end of January 2023.

### Study setting and subjects

The study has been carried out on the adult general population in Ismailia Governorate, Egypt.

### Sampling

#### Sample size

The sample size was calculated using the Epi Info program version 7.1 created by the Centers for Disease Control and Prevention [9]. The confidence level was set at 95%, with a prevalence of awareness of CC in the general population at 90% [10]. The estimated sample size was 135 participants. To improve the generalizability, we added 10% to compensate for non-response. The link to the questionnaire was activated during the aforementioned duration and a total of 150 responses were received.

#### Sampling technique

A snowball sampling technique was used to recruit the participants from Ismailia Governorate, Egypt. To reach a larger population a web-based survey using an online Google form was sent to the general population of Ismailia Governorate through social media platforms like Facebook and WhatsApp. Participants were asked to share the survey link with others.

### Data collection tool

#### Structured self-administered questionnaire

Data was collected using a structured self-administered questionnaire. Literature on CC was reviewed for developing the questionnaire, and it was adapted from prior studies [10–12]. The link of the Google form was opened from 1 October 2022 to the end of January 2023, when the required sample size was reached.

The main parts of the questionnaire include:

- *Personal data*: It includes questions about gender, age, educational level, marital status, occupation, and residence.
- *Assessment of knowledge about CC*: It includes 11 questions asking about CC knowledge and opinions in the general context such as meaning, causes, and importance.
- *Assessment of knowledge about the impact of CC*: It includes 4 questions concerning participants’ knowledge about CC’s impact on health and the environment.

All questions on CC knowledge had responses using various Likert-type answers such as Yes, No, or Don’t Know. Yes, the response was coded 1 and no or do not know were coded 0. For some questions, the responses were options of possible answers to this question and only one response is correct. This correct answer was coded 1 and the wrong answers were coded 0. Responses for questions in the knowledge sections were summed up for each participant. The maximum score was determined and the score percentage was calculated by dividing the participants’ score by the total score. The knowledge score was categorized as not knowledgeable if the percentage score was less than 70% and knowledgeable if it was 70% or more [8].

- *Assessment of attitudes regarding CC*: Three questions were used to assess the attitudes of participants towards CC. The CC attitude questions used Likert-type responses with rating scales from 1 to 3, where 1 = disagree, 2 = undecided, and 3 = agree.

To translate the adapted original English questionnaire, we used the forward–backward translation approach. The questions were translated into Arabic by two language experts. Another two language experts conducted the back-translation into English.

#### Validity and reliability of the questionnaire

To ensure face and content validity, the questions were revised by three experts in public health to confirm that the questions adequately covered the study objectives and to ascertain the understandability of the questions. The required changes were carried out. A pilot study was conducted on 20 participants to test the clarity of the questionnaire. Based on the result of this pilot study, three questions were changed, and one question was omitted. Data from the pilot study was not included in the final analysis. For reliability, Cronbach’s alpha coefficient was calculated as 0.830, indicating the reliability of the questionnaire.

### Statistical analysis

The responses of participants were collected in an Excel spreadsheet, and then imported to the statistical package of the social science statistical program software version 26 (SPSS Inc., Chicago, USA). Descriptive statistics were generated in the form of numbers and percentages. The Chi-square and Fisher’s Exact Test were used for bivariate analysis. Age was not normally distributed, as indicated by the Shapiro–Wilk test, so we used the Mann–Whitney U test for its bivariate analysis. Multivariate analysis of factors affecting knowledge level was conducted using logistic regression. The level of significance was set at .05.

## Results

### Socio-demographic characteristics of the studied participants

Nearly half of respondents (51.3%) were males, with an average age of 35.95 ± 14.05 (range 18–79 years). Around 60% (59.3%) were married and 40% had a bachelor’s degree. About 70% lived in urban areas and 68.7% were employed (Table 1).

**Table 1:** Socio-demographic characteristics of the studied adult general population in Ismailia Governorate, Egypt, (2022-2023)

Socio-demographic characteristics (*n* = 150)	No.	%
**Gender**		
Male	77	51.3
Female	73	48.7
**Age** range	18–79 years	
Mean ± SD	35.95 years ± 14.05	
**Age groups(years)**		
≤ 25 years	32	~ 21.3
26-	42	28.0
37-	42	28.0
48-	14	~ 9.3
59-	9	6.0
≥ 70 years	11	~ 7.3
**Marital status**		
Single	51	34.0
Married	89	59.3
Divorced or widow	10	6.7
**Educational level**		
Read and write/ Elementary	16	~ 10.7
Preparatory	6	4.0
Secondary / Diploma	37	~ 24.7
Bachelor	61	~ 40.7
Postgraduate	30	20.0
**Residence**		
Rural	46	30.7
Urban	104	69.3
**Occupational status**		
Student	23	15.3
Not working	15	10.0
Working	103	68.7
Retired	9	6.0

*SD* Standard deviation

### Knowledge and opinions of the studied participants about CC, its causes, and impact

Concerning CC knowledge, 75.3% reported knowing about it. However, only 60.7% of participants correctly identified its meaning. The mean knowledge score was 27.4 ± 14.42. Regarding the knowledge status of participants, 60% were considered knowledgeable (The total score percent was ≥ 70%) and 40% were not knowledgeable (< 70%) (Table 2).

**Table 2:** Knowledge and opinions of the studied participants about climate change Ismailia Governorate, Egypt, (2022-2023) (*n* = 150)

Knowledge and opinions	No.	%
**Did you know that there is a phenomenon called climate change?**		
No	37	24.7
Yes	113	75.3
**In your opinion, what does climate change mean?**		
Long-term changes in temperatures and weather patterns	91	60.7
Increased concentrations of heat-trapping gases in the atmosphere	16	10.7
Increased ozone depletion	12	8.0
Don’t know	31	20.7
**Have you personally felt visible effects of climate change?**		
No	25	16.7
Yes	125	83.3
**In your opinion, how important is this phenomenon?**		
A dangerous phenomenon that deserves to be addressed	60	40.0
Deserves some attention	32	21.3
Not a priority	16	10.7
It’s not important	15	10.0
I don’t know	27	18.0
**Knowledge of participants**		
**Knowledge score: mean ± SD, median (IQR)**	27.42 ± 14.42, 34.02(22)	
**Knowledge status**		
Not knowledgeable (< 70%)	60	40.0
Knowledgeable (≥ 70%)	90	60.0

With regards to participants’ knowledge about the causes and impact of CC, 76.7% knew that the climate has changed in recent years, and 54% reported that the main cause of CC was increased concentrations of heat-trapping gases in the atmosphere. Moreover, 74% believed that CC affects the environment, with 82% of respondents considering increased summer temperature as the highest negative effect on the environment. More than two-thirds (69.3%) realized that CC affects human health, with 73.3% agreeing that increasing heat stress is the most significant effect (Table 3).

**Table 3:** Knowledge of the participants about the causes and impact of climate change

Knowledge questions about CC (*n* = 150)	No.	%
**Did you know that the climate has changed in the past years?**		
No	23	15.3
Yes	115	76.7
Do not know	12	8.0
**In your opinion, what is the reason for this phenomenon?**		
Human factors only	30	20.0
Natural causes	7	4.7
Both equally cause CC (natural causes and human factors)	60	40.0
The main cause is due to man, but there are natural causes, but few	13	8.7
I do not know	40	26.7
**In your opinion, what is the main cause of CC?**		
Increased concentrations of heat-trapping gases in the atmosphere	81	54.0
Increased ozone hole	19	12.7
An exaggerated phenomenon, and it is a normal natural cycle that is promoted by the industrialized countries	6	4.0
Do not know	44	29.3
**Did you know that CC affects the environment?**		
No	1	0.7
Yes	111	74.0
I don’t know	38	25.3
**In your opinion, which of the following are the negative effects of CC on the environment?**^**a**^		
Increased summer temperature	123	82.0
Increased drop in temperature in winter	91	60.7
Sea level rising	83	55.3
Floods and droughts	93	62.0
Forest fires	106	70.7
Water shortage	90	60.0
Increased insects and pests	93	62.0
Climate migration	74	49.3
**Do you think that CC threatens human physical and psychological health?**		
No	9	6.0
Yes	104	69.3
I don’t know	37	24.7
**In your opinion, which of the following are the negative impacts of CC on health?**^a^		
Affects malaria, infectious diseases, and vector-borne diseases	88	58.7
Increased temperature in summer and thus an increase in heat stress	110	73.3
Increased zoonotic diseases	73	48.7
Increased food and waterborne diseases	80	53.3
Increased malnutrition diseases	83	55.3
Increased mental health problems	94	62.7
Affects access to healthcare	79	52.7

*CC* Climate change

^a^Participants could choose more than one answer

### Knowledge of the studied participants about mitigation and solution of the CC problem

Regarding mitigation of CC, participants believed that increasing afforestation (76.7%), raising public awareness (74.7%), reducing greenhouse gas emissions (74%), complying with environmental laws (74%), and implementing strict government legislation (72%) are methods for mitigation of CC. Around three-quarters (77.3%) of participants believe that governments bear the responsibility when it comes to addressing CC (Table 4).

**Table 4:** Opinion of participants in mitigation of the climate change, Ismailia Governorate, Egypt, (2022-2023)

Opinion questions in mitigation of the CC problem (*n* = 150)	No		Do not know		Yes	
	**No.**	**%**	**No.**	**%**	**No.**	**%**
**In your opinion, which of the following helps mitigate CC?**^a^						
Reducing industrial greenhouse gas emissions and shifting to low-carbon energy sources	14	9.3	25	16.7	111	74.0
Increase afforestation	13	8.7	22	14.7	115	76.7
Increasing public awareness of the phenomenon	12	8.0	26	17.3	112	74.7
Compliance with environmental laws	13	8.7	26	17.3	111	74.0
Strict government legislation and controls	13	8.7	29	19.3	108	72.0
**In your opinion, which entities bear the responsibility of addressing CC?**^a^						
Governments	13	8.7	21	14.0	116	77.3
International organizations	15	10.0	38	25.3	97	64.7
Major industrial countries	13	8.7	37	24.7	100	66.7
Transport companies and factories	15	10.0	43	28.7	92	61.3
Individuals	16	10.7	40	26.7	94	62.7
Research, scientific and academic institutions	14	9.3	33	22.0	103	68.7

*CC* Climate change

^a^Participants could choose more than one answer

Regarding the opinions of participants on methods that can reduce the CC phenomenon, 84.7% of participants supported increasing green spaces, 84% of participants supported making agreements about CC, 83.3% believed that societies and countries should conduct health education, 82.7% of participants favored supporting products that reduce CC, and 79.4% believed that enacting laws and punishing violating individuals and groups will reduce the CC problem. Only 14.7% of participants said that countries can do nothing. Regarding individuals’ role in solving the CC problem, using energy-saving products was the most commonly chosen option by participants (82%), followed by the reuse and recycling of some products (77.3%), agriculture was chosen by 73.3% of participants, rationalizing the use of water was supported by 72.7% of participants and 70% of participants selected the reduction of dependence on means of transportation. Moreover, 13.3% said that people can’t do anything (Table 5).

**Table 5:** Opinions of participants on methods of reducing the CC phenomenon, Ismailia Governorate, Egypt, (2022-2023)

In your opinion, methods to reduce CC phenomenon (*n* = 150)	No.	%
**On the country level: **^**a**^		
Agree with it	126	84.0
Support products that reduce climate change	124	82.7
Enacting laws and punishing violating individuals and groups	119	79.4
Increase green spaces	127	84.7
Health education	125	83.3
Countries can do nothing	22	14.7
**On an individual level: **^**a**^		
Reducing dependence on means of transportation	105	70.0
Reuse and recycling of some products	116	77.3
Reducing wood consumption	91	60.7
Rationalizing the use of water	109	72.7
Increase agriculture and green landscape	110	73.3
Use of energy-saving products	123	82.0
Reduce meat consumption	55	36.7
People can’t do anything	20	13.3

*CC* Climate change

^a^Participants could choose more than one answer

### Attitude of studied participants toward the CC phenomenon

The participants’ responses to the attitude questions indicated that a large percentage of participants (64.7%) believed that CC is a dangerous phenomenon with global implications. Additionally, 64.7% agreed that CC deserves attention and will impact both our environment and future generations. Regarding role of individuals, 64.7% agreed that individual efforts can make a positive difference in addressing CC. Furthermore, 75.3% of participants believed that individuals need training and education on this issue and must contribute to addressing it. With regards to the means of communication to increase awareness and knowledge of CC; most participants agreed that street advertisements (69.3%), social networking sites (86.0%), and television (87.3%), were the most effective means of increasing awareness of CC (Table 6).

**Table 6:** Attitude of participants toward climate change, Ismailia Governorate, Egypt, (2022-2023)

Please indicate your status of agreement with the following statements (*n* = 150)	Disagree		Undecided		Agree	
	**No.**	**%**	**No.**	**%**	**No.**	**%**
**Statements related to the CC phenomenon**^**a**^						
A threat to you and your family	30	20.0	40	26.7	80	53.3
A dangerous phenomenon that poses a threat to the whole world	24	16.0	29	19.3	97	64.7
Global CC will affect our environment in the next ten years	24	16.0	29	19.3	97	64.7
Global CC will affect future generations	26	17.3	27	18.0	97	64.7
The climate is really changing and there has been an extreme rise and fall in temperatures compared to previous years	24	16.0	23	15.3	103	68.7
A phenomenon that deserves attention and addressing it	23	15.3	23	15.3	104	69.3
**Role of individuals in addressing CC**						
Individuals can do nothing to stop global CC	62	41.3	35	23.3	53	35.3
Individual efforts in the face of CC make a positive difference	16	10.7	37	24.7	97	64.7
Individuals must contribute to the confrontation because we are part of the society	12	8.0	31	20.7	107	71.3
Individuals need training and education on this issue	14	9.3	23	15.3	113	75.3
**Means of communication to increase awareness and knowledge of CC**						
Daily newspapers	58	38.7	32	21.3	60	40.0
The radio	57	38.0	27	18.0	66	44.0
Promotional brochures and prints	41	27.3	31	20.7	78	52.0
Lectures and workshops	32	21.3	26	17.3	92	61.3
Festive social activities	28	18.7	27	18.0	95	63.3
Street advertisements	20	13.3	26	17.3	104	69.3
Social networking sites (such as Facebook and Twitter)	4	2.7	17	11.3	129	86.0
Television	4	2.7	15	10.0	131	87.3

*CC* Climate change

^a^Participants could choose more than one answer

### Factors affecting the knowledge status of CC

With regards to the factors influencing the knowledge status, as determined using bivariate analysis; gender, residence, and educational level significantly affect participants’ knowledge (*p* < 0.001). Specifically, females have higher knowledge (64.4%) compared to males. Educational level is also a highly significant factor, with 46.7% of knowledgeable participants having a bachelor’s degree. Residence is another highly significant factor, with 80% of knowledgeable participants living in urban areas (Table S1).

Lastly, the results of logistic regression analysis revealed that gender is a significant predictor for knowledge status as being female is a positive predictor for knowledge status (OR, 3.912; 95% CI, 1.727–8.861; *p* < 0.001). Also, educational level is a significant predictor as having a postgraduate level of education positively affected the knowledge status (OR, 40.566; 95% CI, 3.768–436.771; *p* < 0.002) (Table 7).

**Table 7:** Multivariate logistic regression analysis of factors affecting the knowledge status of the general population (*n* = 150)

Factors	B	OR	*p*-value	95% CI for OR	
				**Lower**	**Upper**
**Gender** (female)	1.364	3.912	0.001^*^	1.727	8.861
**Educational level **(read and write/ elementary is the reference category)			0.003^*^		
Preparatory	0.852	2.344	0.420	0.296	18.582
Secondary / Diploma	-0.035	0.966	0.961	0.245	3.806
University	1.276	3.583	0.061	0.943	13.619
Postgraduate	3.703	40.566	0.002^*^	3.768	436.771
**Residence** (Urban)	0.422	1.525	0.359	0.619	3.756

*OR* odds ratio, *CI* confidence interval

^*^p < 0.05; Nagelkerke R Square = 0.425

## Discussion

In the current study, more than half of the respondents (60%) had adequate knowledge about CC. The participants’ high education level is probably the reason for this result. This result matches an Egyptian study that found that 70% of the participants were aware of CC and its effects [8]. Also, the results are consistent with studies in Oman, and Iran that reported better knowledge of CC among a high proportion of their respondents [13, 14].

The most interesting finding was that a considerable proportion of the participants (60.7%) correctly identified the meaning of CC. This finding is partially aligned with Odonkor et.al. study [15] which found that most Ghanaians (84.4%) correctly understood the concept of global warming. Furthermore, this study demonstrated that more than three-quarters of enrolled participants believed that the climate has changed in the past years. This finding is consistent with previous research, where most participants had observed changes in global climate and increasing temperatures [7, 14, 16].

Just over half of the participants in this study identified rising levels of atmospheric greenhouse gases as the main cause of CC. This revealed a gap in knowledge regarding the recognition of the main cause of CC and subsequently poor awareness of risk identification. A similar result was obtained among residents of Dammam City in Saudi Arabia, where 62% of respondents were knowledgeable about the impact of greenhouse gases on the environment and their contribution to CC [17]. However, A previous study in Egypt found a higher percentage of knowledgeable participants (78.3%) [8]. Another important finding was that three-quarters of the participants recognized that CC negatively impacts the environment. They chose the increase in summer temperature as the most significant effect, followed by forest fires, floods, and droughts. Similarly, research in North America revealed that most Canadians and Americans believed that CC would result in adverse environmental conditions and pose risks to human health [5]. This result aligns with Salem et.al. [8] findings that a majority of participants were aware of the harmful impacts of CC, particularly concerning extreme weather events like heat waves (94.8%).

In this study, a large percentage of participants stated that CC could negatively impact human health (69.3%). The most commonly cited effect, chosen by almost three-quarters of the participants, was heat-related disorders due to high heat stress in summer. This was followed by the potential increase in mental health problems and infectious diseases. This result generally agrees with Odonkor et al. [15] who also found that the majority of the participants (83.4%) believed that CC and global warming could harm human health. Also, our finding are broadly supported by the work of another study in India (2011) which showed that a large proportion of the respondents reported that changing climate has health-related issues. Furthermore, 72.17% of respondents described direct physical hazards of extreme climatic events as the most significant health-related issue of CC, followed by vector-borne diseases (59.62%) [7].

Our study revealed that almost three-quarters of participants favored increasing afforestation, decreasing the emission of greenhouse gases produced by industrial processes, and transitioning to low-carbon energy as a means of mitigation. Using energy-saving products was the most popular choice for individuals to control CC, followed by reducing personal transportation dependence. Likewise, an Omani study has demonstrated that the majority of the respondents believed that decreasing greenhouse gas emissions was the most important method to reduce CC [13]. Also, this finding aligns with an earlier Indian study where 65.21% of respondents suggested that using renewable sources of energy, planting and protecting trees, and using public transport systems would be most effective in tackling CC and preventing further climate change [7].

Nearly two-thirds of the participants in the present study believed that CC is a dangerous phenomenon with global implications for future generations. There are similarities between these attitudes and those described by prior studies indicating that the majority of the enrolled participants were worried about CC and confirmed that CC has serious impacts on human life in the next decades, emphasizing its need for attention [5, 13, 17].

Three-quarters of the participants agreed that people need education on CC. Most participants recommended using television and social networking sites to increase awareness and knowledge about CC. This finding is also in line with that of Pandve et al. [7] who recommended health education regarding CC to prevent further CC, and the use of television to spread information. Also, social media and television were found to be the main sources of information among participants in studies conducted in Egypt and Oman [8, 13].

In the multivariate analysis, a higher educational level and being female were predictive of better CC knowledge. It is not fully understood why females had a better knowledge regarding CC compared to males. One assumption is that females are more worried about their health and that of their family, and thus, they are more motivated to acquire sufficient information about this problem. The results were consistent with McCright (2010) and Jamshidi et.al. (2018). They noticed that females had more knowledge about CC and were more worried about environmental issues than males [14, 18]. Similarly, in the studies of Aydogdu and Yenigün (2016), Kabir et al., (2016), and Salem et.al. (2022), education was significantly associated with CC knowledge. Accordingly, this might point to the importance of enhancement of CC knowledge among less educated groups and in the school and university curricula [8, 16, 19].

### Study limitations

The following limitations should be considered when interpreting the current results: The cross-sectional design of the study does not provide temporality but remains useful for capturing a snapshot and identifying gaps. Further, the study adopted a snowball sampling technique with potential selection bias. Accordingly, we recommend conducting further studies using a probability sampling technique. Although online data collection is an accessible cost-effective method, it is susceptible to survey fraud, imprecise understanding of questions, and sampling bias.

## Conclusion

More than half of the participants were knowledgeable about CC and the role of human activities in CC. Most of the participants support individual actions for mitigating CC and suggested television and social media as the main methods for disseminating information about CC. Females and highly educated participants had significantly greater knowledge of CC. The study encourages the initiation of public campaigns about CC and its mitigating measures and takes advantage of the prominence of television and social media for disseminating valid and reliable information as well as encouraging positive change in attitude and behavior especially for males and people with lower levels of education and those who live in rural areas. These findings can provide baseline information to decision-makers for use as a guide for developing strategies and policies for effective adaptation and mitigation processes. Finally, conducting further large nationwide studies using probability sampling techniques is highly recommended.

### Supplementary Information

Supplementary Material 1: Table S1. Bivariate analysis of risk factors affecting knowledge status of the general population (*n* = 150).

## Data Availability

The datasets used and/or analyzed during the current study are accessible from the corresponding author upon reasonable request.

## Abbreviations

CC: Climate Change
SD: Standard Deviation
