# Supplementary material for: Knowledge and attitude of general population towards climate change and its impact on health in Ismailia Governorate, Egypt
Source: J Egypt Public Health Assoc. 2024 Jul 1;99:15. doi: 10.1186/s42506-024-00162-y (PMC11214935; doi:10.1186/s42506-024-00162-y)
Supplement: Supplementary file 1 — Supplementary Material 1: Table S1. Bivariate analysis of risk factors affecting knowledge status of the general population (n = 150). [file 42506_2024_162_MOESM1_ESM.docx]

**Table S1.** Bivariate analysis of risk factors affecting knowlegde status of the general population (n= 150)

| **Risk factor** | **Knowledge status** | | | | ***P*-value** |
| --- | --- | --- | --- | --- | --- |
|  | **Not knowledgeable** | | **Knowledgeable** | |  |
|  | **No.** | **%** | **No.** | **%** |  |
| **Gender** |  |  |  |  | 0.000* |
| Male | 45 | 75.0 | 32 | 35.6 |  |
| Female | 15 | 25.0 | 58 | 64.4 |  |
| **Age mean (SD)** | 35(15.38) | | 36.58(13.13) | | 0.162^a^ |
| **Marital status** |  |  |  |  | 0.188 |
| Single | 25 | 41.7 | 26 | 28.9 |  |
| Married | 30 | 50 | 59 | 65.6 |  |
| Divorced or widow | 5 | 8.3 | 5 | 5.6 |  |
| **Educational level** |  |  |  |  | 0.000*^b^ |
| Read and write/ Elementary | 11 | 18.3 | 5 | 5.6 |  |
| Preparatory | 3 | 5 | 3 | 3.3 |  |
| Secondary / Diploma | 26 | 43.3 | 11 | 12.2 |  |
| Bachelor | 19 | 31.7 | 42 | 46.7 |  |
| Postgraduate | 1 | 1.7 | 29 | 32.2 |  |
| **Residence** |  |  |  |  | 0.001* |
| Rural | 28 | 46.7 | 18 | 20 |  |
| Urban | 32 | 53.3 | 72 | 80 |  |
| **Occupational status** |  |  |  |  | 0.402 |
| Student | 12 | 20 | 11 | 12.2 |  |
| Not working | 6 | 10 | 9 | 10 |  |
| Working | 37 | 61.7 | 66 | 73.3 |  |
| Retired | 5 | 8.3 | 4 | 4.4 |  |

** Significant level p value < 0.05, ^a^ Mann Whitney U test, ^b^ Fisher's Exact Test.*
